# Frequency of Hypoalbuminemia and In-Hospital Mortality in Acute Ischemic Stroke Patients Presenting at a Tertiary Care Hospital, Hyderabad

**DOI:** 10.7759/cureus.14256

**Published:** 2021-04-02

**Authors:** Faheem Shaikh, Fida H Shaikh, Sultan A Chandio

**Affiliations:** 1 Medicine, Isra University Hospital, Hyderabad, PAK; 2 Medicine, Shaheed Mohtarma Benazir Bhutto Medical University, Larkana, PAK

**Keywords:** acute ischemic stroke, hypoalbuminemia, in-hospital mortality

## Abstract

Background

The aim of this study was to determine the frequency of hypoalbuminemia and in-hospital mortality in acute ischemic stroke patients at a tertiary care hospital in Hyderabad.

Methodology

This was a prospective observational study conducted at the Department of Medicine, Isra University Hospital, Hyderabad, from February 17, 2017 to August 18, 2017. A total of 196 consecutive cases of acute ischemic stroke were included. Hypoalbuminemia was defined as serum albumin of <3.5 mg/dL. In-hospital outcome in terms of survival or death within seven days of admission was assessed and recorded. Data were analyzed using SPSS, version 20.0. (IBM Corp., Armonk, NY, US). Chi-square test was applied, and p-value of ≤0.05 was considered significant.

Results

Out of the 196 acute ischemic stroke cases, 146 (74.5%) were males and 50 (25.5%) were females. The mean age was 49.31 ± 10.46 years. A total of 90 (45.9%) cases had hypoalbuminemia. Out of these 196 cases, 22 (11.2%) expired within seven days of presentation of acute ischemic stroke, and out of these 22 expired cases, 18 (81.8%) had hypoalbuminemia. In-hospital mortality was found to be strongly associated with hypoalbuminemia (p < 0.001).

Conclusions

Frequency of hypoalbuminemia was significantly higher in ischemic stroke patients and was found to be associated with in-hospital mortality, warranting monitoring at regular intervals, as well as recognizing and treating it early for risk stratification.

## Introduction

Stroke is the third most common cause of death and one of the leading causes of disability in both developed and developing countries. The World Health Organization (WHO) has that estimated 5.5 million people died of stroke in 2002, and roughly 20% of these deaths occurred in South Asia [1-3]. A number of risk factors are recognized in the pathophysiology of stroke, such as hypertension, cigarette smoking, hyperlipidemia, and diabetes [1-5]. In Pakistan, it is reported that 72% of stroke cases are due to cerebral infarction while 28% are due to intra-cerebral bleed [4].

Albumin is mainly synthesized in the liver and classified as a non-glycosylated plasma protein. It is a vital component of the blood stream, is involved in the transport of small molecules, is used as a co-factor in different essential pathways, and contributes as a major force in limiting fluid leakage from vascular compartment into the interstitium [6,7]. Patients having stroke with hypoalbuminemia at the time of admission in the hospital are prone to develop infection and poor functional outcome, resulting in an increased risk of mortality [7,8]. Low level of serum albumin is linked with malnutrition, chronic inflammatory diseases representing a negative acute-phase protein, and it decreases in concentration during tissue injury and sepsis [8-10]. Albumin deficiency is often found in hospitalized patients, and is reported in up to 19% of stroke patients [9,11].

Stroke by itself is a major determinant of early in-hospital mortality in patients with acute stroke while hospital-acquired complications worsen the outcome later in the course of the disease [12]. Although serum albumin plays essential role in numerous vital functions of the human body, it also offers neuroprotective effects [13]. It is also believed that low albumin level at admission is found to be associated with poor morbidity [11,13]. Several experimental studies have shown that human albumin therapy significantly ameliorates neurological function, noticeably decreases the volume of cerebral infarction, and abolishes brain swelling in animal models with acute stroke [10,14,15]. Moreover, patients with low albumin levels may also have co-morbid chronic illnesses or neurological conditions that can halt their recovery from stroke [16-18]. This study aimed to investigate the frequency and clinical significance of hypoalbuminemia in acute ischemic stroke patients. Data from this study can help resolve the disparity of variable frequencies in view of demographic, cultural, socioeconomic, and co-morbid conditions and help in establishing the local perspective.

## Materials and methods

This was a prospective observational study carried out at the Department of Medicine, Isra University Hospital, Hyderabad from February 17, 2017 to August 18, 2017. A total of 196 consecutive cases of acute ischemic stroke were included in this study over the specified period. Institutional Ethical Review Board approved the study design for data collection and its use for research purposes. Patients not consenting or having chronic diseases such as malnourishment (generalized loss of muscle mass with or without edema, body mass index [BMI] of <18.5 kg/m^2^, and history of recurrent infections), hypothyroidism, chronic kidney disease, chronic liver disease, and those taking oral contraceptive pills or statins were excluded from the study. Informed consent was obtained from all cases for inclusion in this study and using their data for research. History and physical examination were recorded in all cases. Computed tomography (CT) scan at admission was done by the radiologist with over five years of experience. Physical examination was done to assess muscle power, aphasia, balance, and Glasgow Coma Scale (GCS). The cases were labeled as having ischemic stroke according to predefined operational definitions. Blood samples were drawn in a sterile manner from peripheral veins and collected in specific tubes for the measurement of serum albumin level at the time of admission. In-hospital outcome in terms of survival or death within seven days of admission was assessed and recorded. Data were analyzed using SPSS, version 20.0. (IBM Corp., Armonk, NY, USA). Mean and standard deviation were calculated for continuous variables, and frequency and percentages were calculated for categorical variables. Univariate and multivariate analysis for in-hospital mortality were performed using Chi-square test and multivariate logistic regression analysis, and p-value of ≤0.05 was considered significant.

Operational definition

Acute ischemic stroke was defined as a sudden onset of focal neurological deficit persisting for more than 24 hours. A focal neurologic deficit was defined as a constellation of symptoms and/or signs whose cause could be localized to an anatomic site in the central nervous system, with or without a drop in GCS. Ischemic stroke was diagnosed with the radiological evidence on non-contrast CT scan of the brain at the time of admissions as hypodense area of a particular vascular territory or magnetic resonance imaging of the brain, which was suggestive of acute ischemic stroke related to the focal neurological deficit. Early features of infarction on CT scan were also taken in consideration: hyperdense middle cerebral artery sign, loss of gray-white matter differentiation, hypoattenuation of deep nuclei, cortical hypodensity with associated parenchymal swelling, and resultant gyral effacement.

Hypoalbuminemia was defined as serum albumin <3.5 mg/dL. In-hospital mortality was defined as death of the patient within seven days of presentation of acute ischemic stroke. The patients with hypertension were known hypertensive and were on treatment taking medicines regularly as per the physician’s advice for at least seven days per week. Those who were known type II diabetes mellitus patients and were on treatment taking medicines regularly as per the physician’s advice for at least seven days per week were considered as patients having diabetes mellitus type II. The cases with dyslipidemia were considered as those who were on treatment taking medicines regularly as per the physician’s advice for at least seven days per week and having any component of lipid profile deranged, e.g., cholesterol >200 mg/dL, triglyceride >150 mg/dL, low-density lipoprotein >100 mg/dL, or high-density lipoprotein <40 mg/dL. Those who smoked at least five cigarettes a day for at least one year were considered as smokers. Serum hemoglobin <12 mg/dL in males and <11 mg/dL in females was regarded as anemia.

## Results

A total of 196 acute ischemic stroke cases who met the eligibility criteria were enrolled in this study. The mean age of the cases was 49.31 ± 10.46 years. Out of these 196 cases, 146 (74.5%) were males and 50 (25.5%) females. The majority of the cases belonged to the fifth decade of life. Among these study cases, 160 (81.6%) had hypertension, 90 (45.9%) had hypoalbuminemia, 79 (40.3%) had type II diabetes mellitus, 75 (38.3%) were smokers, 70 (35.7%) had a history of ischemic heart disease (IHD), 57 (29.1%) had dyslipidemia, and 22 (11.2%) had anemia.

Of these 196 cases, 22 (11.2%) expired within seven days of presentation of acute ischemic stroke. Out of these 22 cases who expired, 90.9% were smokers, 81.8% had hypoalbuminemia, 72.7% had history of IHD, 54.5% had hypertension, and 45.5% had type II diabetes mellitus. Among the cases who expired, majority had a BMI of <30kg/m^2^ (72.7%) and mostly belonged to low and middle socioeconomic status (95.45%). The different variables are listed in Table 1 in relation to in-hospital outcome and hypoalbuminemia. In-hospital mortality was found to be strongly associated with hypoalbuminemia, with a p-value <0.001.

**Table 1:** Characteristics of cases presenting with acute ischemic stroke. P-values are based on Chi-square test

Variable		Survived	Expired	P-Value
Age	<40 years	40 (23%)	4 (18.2%)	<0.001
	41-60 years	73 (42%)	1 (4.5%)	
	>60 years	61 (35.1%)	17 (77.3%)	
Gender	Male	138 (79.3%)	8 (36.4%)	<0.001
	Female	36 (20.7%)	14 (63.6%)	
Hypoalbuminemia	Yes	72 (41.4%)	18 (81.8%)	<0.001
	No	102 (58.6%)	4 (18.2%)	
Diabetes mellitus type II	Yes	69 (39.7%)	10 (45.5%)	0.38
	No	105 (60.3%)	12 (54.5%)	
Hypertension	Yes	123 (70.7%)	12 (54.5%)	0.09
	No	51 (29.3%)	10 (45.5%)	
Dyslipidemia	Yes	54 (31%)	3 (13.6%)	0.06
	No	120 (69%)	19 (86.4%)	
History of ischemic heart disease	Yes	54 (31%)	16 (72.7%)	<0.001
	No	120 (69%)	6 (27.3%)	
Anemia	Yes	20 (11.5%)	2 (9.1%)	0.53
	No	154 (88.5%)	20 (90.9%)	
Smoker	Yes	55 (31.6%)	20 (90.9%)	<0.001
	No	119 (68.4%)	2 (9.1%)	
Body mass index	<30 kg/m^2^	123 (70.7%)	16 (72.7%)	0.53
	>30 kg/m^2^	51 (29.3%)	6 (27.3%)	
Socioeconomic status	Lower class	102 (58.6%)	9 (40.9%)	0.27
	Middle class	65 (37.4%)	12 (54.5%)	
	Upper class	7 (4%)	1 (4.5%)	

Hosmer and Lemeshow test Chi-square value for multivariate logistic regression for in-hospital mortality was 14.542, with p-value of 0.042 at seven degrees of freedom. The only statistically significant factors were history of IHD and hypoalbuminemia with p-values of 0.002 and 0.021, respectively.

## Discussion

The precise prediction of outcome in cases with acute stroke is beneficial for selecting specific management plans, employing accurate therapeutic measures, anticipating discharge from the hospital, and foretelling the required rehabilitation and community support [19], especially in resource-limited areas such as Pakistan. Low levels of serum albumin have been studied as a prognostic factor for clinical outcomes and mortality not only in patients with stroke but in various other diseases as well [20]. Numerous studies have highlighted the impact of hypoalbuminemia on mortality and its therapeutic use in acute ischemic stroke [7,21].

This study revealed hypoalbuminemia in 45.9% among the enrolled cases. Of the patients who expired in the hospital, 81.8% were found to have hypoalbuminemia. These findings are consistent with the study conducted by Butt et al. at Islamabad, who reported hypoalbuminemia in 41.6% of their cases suffering from acute ischemic stroke, but only 28.85% of their hypoalbuminemia cases died [16]. Vahedi et al. evaluated the relationship between hypoalbuminemia and in-hospital mortality in patients with acute stroke and found the prevalence of hypoalbuminemia in 43.8% of ischemic stroke patients, which is comparable to the findings of the present study. They reported mortality in 75.8% of the hypoalbuminemia cases [12]. Dziedzic et al. also reported almost comparable frequency of hypoalbuminemia in their study [13]. Sani Abubakar and co-workers reported low admission serum albumin as an independent factor in determining the poor outcome in the cases under their study [17]. Famakin et al. studied a biracial cohort of patients with acute stroke presented at 34 hospitals in the state of Georgia. They identified hypoalbuminemia as an independent prognostic factor of predicting mortality with a frequency of 23.3% [8].

Protein malnutrition on admission may influence prognosis of 8-30% of stroke patients, which significantly correlated with the overall nutritional status of the patients at the time of hospitalization [9]. Davis et al. assessed the impact of undernutrition as an independent predictor of poor stroke outcome and found hypoalbuminemia in 16.2% of the cases with acute ischemic stroke [18]. The present study was conducted at a center where most of patients come from rural areas. The majority of the cases in this study were non obese (70.91%), and most of them belonged to the lower socioeconomic class (56.63%), which emphasizes the fact that limitation of resources in a society leading to pre-morbid protein malnutrition that ultimately worsens the outcome of that particular disease.

The observed differences in the frequency of hypoalbuminemia in this study and from some of the previously reported studies can be attributed to methodology, duration and length of follow-up, local demographics, and patient selection such as cases with ischemic and hemorrhagic stroke versus only ischemic stroke.

It is vital to evaluate the role of serum albumin as a pointer of clinical outcomes in vascular diseases. Not only in stroke but serum albumin has been linked with unfavorable vascular events in patients with cardiac and renal diseases [20]. Serum levels of albumin are maintained by factors affecting protein synthesis, breakdown, leakage to the extravascular space, and overall nutritional status.

Albumin levels have long been used as a measure of health and disease. This may be due to malnutrition, catabolism, and/or prior underlying disease processes, e.g., renal or hepatic insufficiency, decompensated heart failure, and malignancy. This small but systematic study has some important implications but additional studies are required to address the importance of monitoring albumin level and to determine the effect of traditional cardiovascular disease risk factors and their impact on outcome in stroke patients.

This study has some limitations such as short duration of study, limited number of cases from a single center, and duration of follow-up. Moreover, mortality assessment was short which could have been up to 30 days, and other premorbid chronic illnesses were not included in the assessment.

## Conclusions

The study shows the importance of albumin level while treating stroke patients. The frequency of hypoalbuminemia was found to be significantly higher in ischemic stroke patients in our study. Although our population was from a lower-middle-income country with already prevalent hypoalbuminemia, a significant association of hypoalbuminemia with in-hospital mortality cannot be sidelined. These findings warrant that there should be monitoring of albumin levels at regular intervals to recognize these issues early and for optimum treatment. There is also a need for a larger study at national and regional levels to judge the significance of hypoalbuminemia in different populations. Furthermore, it is necessary that the government should invest resources to inform the population about the significance of hypoalbuminemia and take measures to correct it.
